# Are Randomized Controlled Trials the (G)old Standard? From Clinical Intelligence to Prescriptive Analytics

**DOI:** 10.2196/jmir.5549

**Published:** 2016-07-06

**Authors:** Sven Van Poucke, Michiel Thomeer, John Heath, Milan Vukicevic

**Affiliations:** ^1^ Department of Anesthesiology, Critical Care, Emergency Medicine, Pain Therapy Ziekenhuis Oost-Limburg Genk Belgium; ^2^ Department of Pneumology Ziekenhuis Oost-Limburg Genk Belgium; ^3^ RapidMiner Shanghai China; ^4^ Faculty of Organizational Sciences University of Belgrade Belgrade Serbia

**Keywords:** randomized controlled trials, data mining, big data, predictive analytics, algorithm, modeling, ensemble methods

## Abstract

Despite the accelerating pace of scientific discovery, the current clinical research enterprise does not sufficiently address pressing clinical questions. Given the constraints on clinical trials, for a majority of clinical questions, the only relevant data available to aid in decision making are based on observation and experience. Our purpose here is 3-fold. First, we describe the classic context of medical research guided by Poppers’ scientific epistemology of “falsificationism.” Second, we discuss challenges and shortcomings of randomized controlled trials and present the potential of observational studies based on big data. Third, we cover several obstacles related to the use of observational (retrospective) data in clinical studies. We conclude that randomized controlled trials are not at risk for extinction, but innovations in statistics, machine learning, and big data analytics may generate a completely new ecosystem for exploration and validation.

## Introduction

Despite the accelerating pace of scientific discovery, the current clinical research enterprise does not fully address daily clinical questions such as “what is the most adequate course of action for a particular patient, under these conditions, in this phase of the illness?” [1]. From a clinician’s perspective, the most abundant information available for decision making is based on observation and experience [2]. With the accumulation of large amounts of health-related data, the methods for therapeutic effect quantification have been rapidly evolving and are driven by recent innovations in statistics, machine learning, and big data analytics [3]. Recent technology allows the use of (near) real-time clinical decision support tools, enabling the quantification and prioritization of unanswered clinical questions in the absence of published evidence [4].

Despite the abundance of data available, fitting data to a model to explain observations might be plausible and appear to be in agreement with clinical experiences, but the derivation of natural laws or theories cannot be justified. From an epistemological point of view (Karl Popper), science should strive to describe simple and logical theoretical systems that are testable before enabling any predictions [5]. Classically, deductive science begins with a hypothesis or theory and proceeds to derive possible conclusions and statements. With the introduction of precision medicine as an emerging approach for disease treatment and prevention, the question arises whether simple and logical theoretical systems are the only choice for predictive analysis of complex, high-dimensional data from a multimorbidity patient population [6].

Various methods have been presented to predict future outcomes or to forecast trends using observational data [7]. Observational data research might seem attractive because of lower cost and time consumption, but it is mostly considered inferior to prospective research. In the big data and Internet of things era, “observational” data are abundant and could be considered a historical footprint, valuable for training and testing models from which performance can be quantitatively assessed using new data input [8].

The aim of this viewpoint paper is to highlight some innovations in statistics, machine learning, and big data analytics, and to confront them with the current gold standard test used in clinical trials: the randomized controlled trial (RCT). Therefore, we discuss this in three sections—challenges and shortcomings of RCTs, potential of observational studies with big data, and challenges and difficulties of observational (retrospective) data for clinical studies.

## The Challenges and Shortcomings of RCTs

RCTs were introduced in medicine more than half a century ago [9]. The trial is initiated by a null hypothesis that there is no decisive evidence that the intervention or drug being tested is superior to existing treatments. In prospective RCTs, the investigators conceive and design the trial, recruit participants, and collect baseline data, before the participants have developed any of the outcomes of interest. Individuals are selected from a population to estimate characteristics of the entire population. The intervention is randomly assigned after participants have been assessed for eligibility and recruitment, but prior to the intervention under study. When properly designed, RCTs can isolate confounding factors and allow researchers to identify causal effects between input and observed phenomena. This makes RCTs the gold standard for evidence-based medicine (EBM) [10]. The Framingham Heart Study is a historical example of a large, productive prospective cohort study [11].

In contrast, it is widely acknowledged that evidence from RCTs frequently rests on narrow patient inclusion criteria, hindering generalization to real clinical situations [12]. As such, RCTs do not ensure the translation of their results into tangible benefits to the general population [13]. Additionally, it is often unclear which assumptions are part of the hypothesis. Frequently, researchers end up with central tendencies from a group of individuals, a measure that is often not representative of an individual patient.

Limitations of RCTs or suboptimally designed RCTs are at times overlooked or ignored [14]. When RCTs lack methodological rigor, the results must be interpreted cautiously [15,16]. Furthermore, the cost and duration of RCTs may be prohibitive, delaying the acceptance of new treatment modalities [17]. The outcome of interest in RCTs should also be common; otherwise, the number of outcomes observed becomes too small for statistical meaningfulness (indistinguishable from the cases that may have arisen by chance).

Additionally, certain interventions might not be suitable to be explored by RCTs because of ethical considerations. Likewise, when an intervention becomes widespread, clinicians are unwilling to experiment with alternatives. For instance, the impact of timing of cardiopulmonary resuscitation on cerebral and myocardial functional recovery cannot be investigated with controlled trials. However, such studies can be designed using techniques such as propensity score analysis and stratification based on big data [18,19].

With the aging of the population, an increasing percentage of patients have multiple comorbidities, which are routinely excluded from RCTs. In contrast, big data from electronic medical records provide information from real-world settings [19]. Research based on these data might be more applicable to patients encountered in daily practice.

Even with a well-designed and successfully conducted RCT, many clinical questions are unanswered, because results from RCTs might not be suited to each individual patient. This problem is the main focus of personalized and precision medicine [6]. An obvious example is that over the past few decades, perioperative management has improved in safety, resulting in lower incidences of major perioperative complications (<1% to 3%), such as perioperative stroke or death. Nonetheless, even events with a 1% incidence rate would affect 2 million people each year worldwide. These devastating complications are hardly studied in RCTs, as their low incidences would require inclusion of significant numbers of patients [20]. Big data analytics might facilitate research for these rare end points, thereby potentially opening opportunities for improving clinical practice [21].

In the last two decades, EBM attempted to address the limitations of RCTs. EBM is commonly defined as “the conscientious, explicit, and judicious use of current best evidence in making decisions about the care of individual patients” [22]. The purpose of EBM is to provide a stronger scientific foundation for clinical work, so as to achieve consistency, efficiency, effectiveness, quality, and safety in medical care. The theoretical ideal of EBM, where every clinical question would be based on meta-analysis and systematic reviews of multiple RCTs, faces multiple limitations. An early example of EBM can be found in the British Thoracic Society’s 1990 asthma guidelines, developed through consensus and based on a combination of randomized trials and observational studies [23].

Two decades of enthusiasm could not prevent some from arguing that the EBM movement is in crisis, for many reasons [2]: (1) the evidence-based “quality mark” has been misappropriated by vested interests, (2) the volume of evidence, especially clinical guidelines, has become unmanageable, (3) statistically significant benefits may be marginal in clinical practice, (4) inflexible rules and technology-driven prompts may produce care that is *management driven* rather than *patient centered*, and (5) evidence-based guidelines often map poorly to complex multimorbidity.

It is remarkable that recent advances in analytics are not mentioned in any “strength of evidence” rankings [24]. This closely resembles the plea from Angus Deaton, the 2015 Nobel prize winner in economic sciences, for more modesty in what randomized trials can offer, fulminating against a one-size-fits-all mentality [25].

## Potential of Observational Studies With Big Data

The burden of chronic diseases is rapidly increasing worldwide, triggering a paradigm shift from delayed interventional to predictive, preventive, and personalized medicine [26,27]. Success stories of the big data paradigm and data mining led to broader recognition of the potential impact and benefits (both human and economic) in health care. In 2012, the worldwide amount of digital health care data was estimated to be around 500 petabytes, expected to reach 25,000 petabytes in 2020, of which approximately 80% is unstructured [28].

The explosion in data has opened a multitude of opportunities for improving health care in general by the design of data-driven models for different tasks: (1) in public health: prediction of admission rates, epidemics, hospital capacities, etc, (2) early risk prediction for mortality, hospital readmission, treatment efficacy, etc, (3) for chronic disease control: drug dosage optimization, therapeutic adherence, etc, (4) in diagnostics: decision support systems in medical imaging, etc.

Predictive modelling in a clinical context, where data are collected, a statistical model is formulated, predictions are made, and the model is validated (or revised) as additional data become available, could become the key for tailoring medical treatment to individual characteristics of each patient (precision medicine initiative [6]).

A recent report on the potential of learning health care systems suggested that the RCT is not dead, but rather that other methodologies will be required if we are to bridge the evidence gap in modern medicine [29]. Observational studies can deliver useful results quickly, at lower cost, and do not put patients at risk through experimental exposure. The development of electronic health records and rigorous outcomes measurement offers the potential to accelerate the use of observational research. This may require a paradigm shift in education and research.

**Figure 1:**
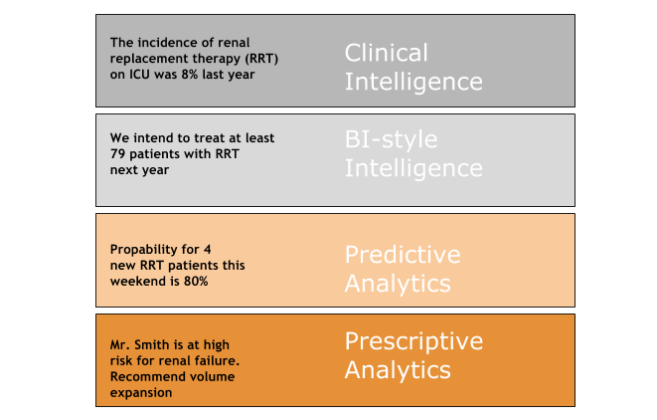
From clinical intelligence to prescriptive analytics. BI business intelligence; ICU: intensive care unit.

Retrospective data are historically assessed by descriptive statistical analysis, resulting in clinical intelligence (Figure 1). Predictive analytics differs from clinical intelligence and business intelligence-style intelligence in its use of models—models that capture and represent hidden patterns and interactions in the data.

Clinical decisions, once exclusively guided by *experience* (wisdom generated from qualitative retrospective analysis) and *retrospective clinical intelligence* (wisdom from quantitative retrospective analysis), can now be upgraded by knowledge of predictive and prescriptive analytics, predicting future events on the individual patient level (Figure 1).

Big data is defined as high-volume, high-velocity, high-variety, and high-veracity information assets, requiring new forms of processing to enhance decision making, insight discovery, and process optimization [30]. Cutting-edge big data technologies allow for integration and scalable analytics of heterogeneous medical data. Additionally, recent computational and mathematical advances have enabled effective usage of machine learning and data mining methods for uncovering hidden relationships between different parameters and clinical outcomes [13]. This evolution is considered one of the main factors in the development of predictive, preventive, and personalized medicine. Big data might increase the relatively low ratio of screened to enrolled patients of RCTs, optimizing the generalization of results from research in routine clinical practice (external validity).

Data availability in clinical medicine can be seen as both wide (from large populations) and deep (a large amount of data per patient). Wide data allow for analytics of various trends in public health care (eg, the number of admissions per disease or hospital) and can be used in quality indicators for hospitals (eg, readmission rates), newly introduced drugs, or health campaigns. In other words, wide secondary data provide the essential raw material for key operations in health care. Plans and priorities of governmental health departments and clinical decision making based on historical disease characteristics both depend on secondary data. For example, virtually every basic-science grant application for severe sepsis research contextualizes the proposed work with national-scale epidemiology derived from administrative records [27,31]. Policy concerns about health care overuse in the intensive care unit, such as excessive end-of-life spending and unexplained geographic variation in intensive care unit use, depend on secondary data analyses [32,33]. Much of our understanding of racial or ethnic and insurance-based disparities, as well as the value of critical care, derives from secondary data analyses [34].

Directly related to the exploration of wide data, initiatives were promoted for collecting, integrating, and making publicly available these data for analyses. One of the largest open databases of this kind is the State Inpatient Databases, a US Agency for Healthcare Research and Quality Healthcare Cost and Utilization Project [35]. The State Inpatient Databases (2001–2010) include about 330 million inpatient discharges from 46 US states. These data track all hospital admissions at the individual level, and track diagnostic and procedural data based on *International Classification of Diseases, Ninth Revision, Clinical Modification* coding. Additionally, demographics and administrative data of each admission are tracked (eg, sex, age, month of admission, length of stay, and total charges in US currency). Opening up these data initiated many research efforts in health care predictive analytics as published on websites from the US National Information Center on Health Services Research and Health Care Technology and others.

However, wide data are not the best information source to generate clinically relevant research at the patient level (eg, mortality risk, evaluation of effectiveness of procedures), because these data are in most cases generated for administrative and reimbursement purposes, and are not sufficiently detailed to describe complex medical states and outcomes for a unique patient.

Deep data, on the other hand, provide a higher level of temporal details from each patient, on multiple scales (eg, genomics, proteomics, drugs, laboratory tests, comorbidities, symptoms). When analyzed properly, such data have the potential to provide valuable clinical insights and could change practice in fundamental ways, improving outcomes for patients [6]. A good example is the reevaluation of the use of pulmonary artery catheters, once a ubiquitous feature of the treatment of nearly every medical intensive care unit patient, but this use was reinvestigated with a clever reanalysis of a clinical trial [36].

The importance of opening deep data for analytics is recognized widely. One of the most popular and most detailed data sources available is the Multiparameter Intelligent Monitoring in Intensive Care (MIMIC) clinical database, which contains data on 58,976 intensive care unit admissions (medical, surgical, coronary care, and neonatal), for over 48,000 distinct patients admitted to Beth Israel Deaconess Medical Center (Boston, MA, USA) from 2001 to 2012 [37]. The MIMIC-III database contains highly detailed and heterogeneous data (laboratory tests, vital signs, symptoms, medical imaging, notes, waveforms, etc). The data in the MIMIC-III database are available to other researchers and there are no privacy concerns, promoting reproducibility of research. Opening this database yielded many promising research efforts [38,39].

## Challenges and Difficulties of Observational (Retrospective) Data for Clinical Studies

Observational studies look at medical events from some time point in the past and examine exposure to a suspected risk or treatment in relation to an outcome established at the start of the study. There are several challenges opposing the quick and easy development of predictive models with good performance, in particular for complex clinical problems.

This results in a large gap between potential and actual data usage [27,31]. Retrospective databases pose a series of methodological challenges, some of which are unique to this data source [40].

### Correlation Does Not Imply Causation

One of the major obstacles to full applicability of predictive analytics in real-life clinical practice (and distrust of observational studies) is the credibility of the evolved patterns (models). Although modelling enables quantification of correlation on large data sources, correlation in most cases does not imply causation (even with significant correlations identified). Two major fallacies have been described in this respect: *cum hoc ergo propter hoc*, Latin for “with this, therefore because of this,” and *post hoc ergo propter hoc*, Latin for “after this, therefore because of this.” The main cause of misleading conclusions based on identified correlations is incorrect sample or feature selection, which leads to neglect of actual confounders. Namely, retrospective studies are often conducted on large data samples, but these samples are not described with all potential confounders [41,42]. On the other hand, stratification of a population, leading to homogeneous and well-described groups (eg, the same age group, sex, history of diseases, current health status, and vital signs), also leads to insufficient data quantities because of the complexity of medical phenomena and the large number of potential confounders. So, when a population is carefully selected, in most cases, the lack of data emerges as a problem that prevents the development of accurate and stable predictive models. In these situations, an additional problem arises in identification of real causal relationships: “the curse of dimensionality” or Hughes phenomenon [43]. The curse of dimensionality states that, with a fixed number of training samples, the predictive power reduces as the dimensionality increases, meaning that patterns identified in high-dimensional spaces may occur due to chance. Consequently, an enormous amount of data is needed to ensure that a population is well described by a given sample.

To conclude, in theory it is possible to select an adequate sample and feature space that describes well the medical phenomena that are observed, and eventually could lead to causal relationships and insights. However, finding such samples in retrospective data is very challenging, and this problem has to be addressed adequately when reporting and interpreting predictive results from retrospective studies.

### Fusion of Data Science and Domain Expertise

Even if retrospective studies are well defined (in relation to samples and features) and if the medical community is confident with models and results, successful predictive analytics and application of cutting-edge machine learning algorithms often demands substantial programming skills in different languages (eg, Python or R). This migrates modelling from the domain expert to the data scientist, often missing the necessary domain expertise, and vice versa. Additionally, data analyses are highly creative processes and there are no detailed recommendations for conducting such research. High-level steps for conducting this research is described by the cross-industry standard process for data mining, which is breaking down the life cycle of an analytics project into six phases: business understanding, data understanding, data preparation, modelling, evaluation, and deployment [44]. However, specifications of each problem prevent the development of a standardized analytics process on an operational level. This ultimately leads to the slow development, adoption, and exploitation of highly accurate predictive models, in particular in medical practice, where errors have significant consequences (both human and financial) [45]. Obviously, a close and continuous collaboration between domain experts and data scientists would solve this problem, but this is not always feasible. Many efforts have attempted to overcome this problem in recent research. One of the directions is formalization of domain knowledge through medical ontologies (eg, Disease Ontology [46], SNOMED [47], and for orofacial pain [48]) and integration with data-driven models [49,50]. This approach aims to allow for data-knowledge fusion and to reduce the need for additional specialization of domain experts in data science and vice versa. Another approach is development of visual analytics tools that enable a faster learning curve and powerful analytics that can be conducted by domain experts [45,51].

### Data Heterogeneity and Quality

In particular, deep medical data that could potentially provide meaningful clinical conclusions or new hypotheses is highly heterogeneous: laboratory tests, disease history, comorbidities (multiple diagnoses), medication prescriptions, protein interaction networks, genomic sequences, medical imaging, notes, waveforms, and so on. In addition to different data formats, the data are time stamped, temporal, context dependent, and defined over different levels of granularity. This raises the challenging problem of extracting information and meaningful patterns from all available data sources, even with cutting-edge big data technologies that allow for efficient storage and manipulation of such data and predictive methods that allow for temporal modelling of interdependent data [52]. Various ways have been proposed to address these problems, such as integrating the results of models that are built separately on homogeneous data sources, and mapping between problem (data) spaces and learning models on common data representations [53]. However, each step of these strategies loses information and propagates uncertainty, and thus the potential of big and heterogeneous data is only partially exploited. Additionally, it is essential to interpret the findings in the context of a defined patient population (generalizability). If multiple data sources were used to construct a database, it is important to emphasize whether the necessary linkages between data sources and various care sites have been carried out appropriately, taking into account differences in coding and reporting across sources and timestamping (data linkage). Retrospective data face a renewed interest with the growth of big datasets, as questions arise related to the quality of the data and the source validity. With frequently unknown quality or completeness of the recorded data, “garbage in, garbage out” (or GIGO) is commonly used to describe failures in human decision making due to faulty, incomplete [38], or imprecise data.

### Validation and Reproducibility

Even though many studies have reported cutting-edge performance in predictive modelling on biomedical data, evolved models often show unstable or unconvincing performance when applied outside of the initial experimental setting.

Some of the reasons for this are that validation measures used in experiments are misleading; that specific algorithm implementations and data are not always available; and that experimental settings are not sufficiently described and, thus, the results reported in scientific papers cannot be reproduced in other settings.

Selection of adequate validation measures is highly dependent on the nature of the data for building models. Since most of these datasets have an imbalance between the size of the positive and negative classes, classification accuracy is a meaningless performance measurement. For this reason, other evaluation criteria are used, such as the area under the receiver operating characteristic curve and the area under the precision recall curve. All of these are based on the basic notions of the numbers of true positives, false positives, true negatives, and false negatives [54,55].

Further, in order to realistically estimate model performance in the future (on unseen cases), experimental setups will need perfection and to be protected against overfitting (the situation where a model has good performance on training data but shows poor generalization performance when tested on unseen cases). As discussed before, finding the predictive model best suited to the data at hand is often based on trial and error, and assumes comparisons of multiple models with multiple parameter settings. The number of trials and the complexity of the models positively correlate with the probability of model overfitting.

This is why parameter optimization and multiple model testing should also be monitored using an alternative partition of the data (validation dataset). A common technique to validate a model is either cross-validation or bootstrap validation [56]. Cross-validation is often used to select the optimal level of complexity (maximal predictive power without overfitting).

Other methods focus on estimating heterogeneity in causal effects in experimental and observational studies, and on conducting hypothesis tests of the magnitude of the differences in treatment effects across subsets of the population. These approaches are often tailored to situations with multiple attributes of a unit relative to the number of units observed, and where the functional form of the relationship between treatment effects and the attributes of units is unknown [19].

Finally, the error rate of the model is estimated with the remaining data partition [57]. As such, the testing data represents a realistic assessment of the model’s correctness when applied to new datasets. Additionally, it is utterly important to take special care when selecting data for validation and final model performance evaluation (because models could adapt and generalize well only on a subset of the data, and thus all types of data that are expected in the future have to be present in the final evaluation of the model).

When modelling is done properly, accurate predictive models have the ability to adjust and improve over time. The artificial intelligence resulting from this evolution might have the potential to measure and optimize therapeutic effect and adherence [58].

### Interpretability

In the process of building a useful representation of a system or phenomenon, interpretability (comprehensibility or ability to understand) is often recalled. This is of particular importance in the medical domain because, even with the best diagnostic assessment and highly accurate predictive models, decisions have to be made with caution and with involvement of medical experts. If models are interpretable, medical experts can put information provided by predictive models in their specific context (reducing the danger of potential confounder influence) and get better insights into the reasons for phenomena identified by predictive models. This should eventually lead to making informed decisions and taking a step toward prescriptive analytics. However, there is a clear trade-off between model complexity and model interpretability. Additionally, interpretability is in the eye of the beholder: it is hard to make some objective comparisons between predictive models. Model interpretability is also related to the number of features and the information provided by the features. The number of features is intuitively evident as an interpretability measure. The higher the dimensionality, the more complex it becomes for human beings to analyze the relative impact of features and patterns that are potentially important in making decisions. Therefore, using a reduced set of features might lead to more interpretable models (eg, through backward feature elimination, or forward feature construction). The basic principle of all predictive methods for decreasing the number of features is to extract factors from features, by mapping (transforming) the feature space to a low-dimensional space, while keeping as much of the original variance of the features as possible.

On the other hand, the contextual information provided by the features is important regardless of dimensionality. If a model is based on a limited number of features but the human interpreter considers the model to be a black box, then the model is not interpretable. Interpretability requires more thought on how the results of predictive models help in explaining an underlying phenomenon [59]. Because of this, state-of-the-art predictive algorithms, which often provide highly accurate models (eg, neural networks or support vector machines), are often not considered useful for real-life medical applications. This poses an additional challenge to making highly accurate predictive models based on less-complex and more-interpretable algorithms such as logistic regression, naive Bayes, or decision trees. Unfortunately, interpretability and accuracy are usually concurrent, and this increases the importance of feature selection and construction in predictive modelling processes.

### There Is No Free Lunch

Many predictive algorithms have been developed, but there is no evidence that any algorithm outperforms all others in every situation. Strong support for this claim is given by “no free lunch” theories [60], where researchers demonstrate that no predictive algorithm outperforms others on every dataset, but one can always find an algorithm that is optimal for a dataset. In particular, in health care predictive analytics, the consequences of no free lunch theories are posing a very challenging problem of finding the algorithm best suited to the data at hand. This is directly related to the complexity of medical phenomena, contextual dependency, data heterogeneity, high dimensionality, class imbalance, and so on. For many of these specific problems, a variety of efficient predictive methods have been developed. For example, lasso logistic regression efficiently reduces dimensionality of the initial dataset [61], while preserving or even increasing the predictive performance on unseen data. Support vector machines [62] efficiently avoid overfitting and allow incorporation of domain knowledge by kernel engineering. Neural networks and deep learning methods have the ability to fit high-dimensional data and to model spatiotemporal relations in data [63]. Further, ensemble methods [64] are used to improve the performance of individual algorithms. They have shown many advantages in dealing with a small sample size, high dimensionality, and complex data structures by exploiting the diversity among the models produced. These models can be aggregated from the same model built on different subsamples of data, from different models built on the same sample, or a combination of the previous two techniques. Some popular algorithms from this class are bagging [65], random forest [66], boosting [67], and bootstrap aggregating.

However, all mentioned models have their own cons and there are no theoretical guarantees for a model’s success in a particular application. The problem of finding the best model for a particular dataset is influenced by data preprocessing (feature selection, feature construction, etc). The objective of variable (feature) selection is 3-fold: improving the prediction performance of the predictors, providing faster and more cost-effective predictors, and providing a better understanding of the underlying process that generated the data [68]. This requires feature construction, feature ranking, multivariate feature selection, efficient search methods, and feature validity assessment methods.

### Privacy Concerns

Another problem often considered an obstacle for successful application of predictive analytics in health care is the lack of data. Data can be lacking for several reasons: rare diseases, long and expensive procedures for data collection, and confidentiality of personally sensitive information. Privacy concerns often restrict the potential of sharing the data between institutions and thus building more accurate and reliable models.

However, there are many techniques that could help in overcoming this problem and enable data sharing without fear of identifying patients without their permission. The process of privacy protection starts with traditional anonymization techniques, which map personal and hospital identity into an encrypted form. Additionally, time and duration of hospital visits are usually presented in a relative form (number of days from initial admission), while exact dates are removed. Even though these techniques can substantially reduce the risk of patient identification, the state-of-the-art predictive techniques theoretically can still identify the person based on procedures, diagnoses, and other data that cannot be encrypted if they are a basis for collaborative building and evaluation of predictive models. Thus, privacy of big data is of particular concern. These problems are often successfully solved by secure multiparty computation [69,70], where the sites cooperate to build the global prediction model without sharing the data themselves, and by randomization, where data are additionally masked by adding some controlled noise [71,72].

## Conclusion

By no means is the value of RCTs as a method for scientific experimentation questioned. We are convinced that it is far more reasonable to estimate the therapeutic effects from nonrandomized studies, based on the best available surrogate technology, than to ignore the potential richness of the available data [13]. Nonrandomized data could at least provide indicators of potential causality, ultimately triggering the initiation of randomized experiments.

A changing ecosystem of analytical methods has opened up and become available for exploration and validation. Observational studies could complement RCTs in generating hypotheses, establishing questions for future RCTs, and defining clinical conditions [73]. Drawing conclusions based on biased data or dubious analyses by threats of both external and internal validity should be monitored constantly in big data analysis to guarantee that a study measures what it set out to and that the results can be generalized from the study to the reader’s patients.

As such, the data science community has a huge responsibility to eliminate the fear of using predictive modelling in health care by explaining the concepts of predictive modelling in a setting where humans are the preferred decision makers. Finally, data scientists need to create familiarity with data visualization as a channel for information sharing. Data-driven research incorporates artificial intelligence and machine learning into statistics and supports the recognition of patterns within massive datasets. Validation and interpretation of results is an essential step preceding data visualization.

## Abbreviations

EBM: evidence-based medicine
MIMIC: Multiparameter Intelligent Monitoring in Intensive Care
RCT: randomized controlled trial
